# A machine learning approach for predicting radiation-induced hypothyroidism in patients with nasopharyngeal carcinoma undergoing tomotherapy

**DOI:** 10.1038/s41598-024-59249-3

**Published:** 2024-04-10

**Authors:** Ke-Run Quan, Wen-Rong Lin, Jia-Biao Hong, Yu-Hao Lin, Kai-Qiang Chen, Ji-Hong Chen, Pin-Jing Cheng

**Affiliations:** 1 Department of Radiation Oncology, Xiangtan Central Hospital, Xiangtan, 411100 Hunan China; 2 Department of Radiation Oncology, Clinical Oncology School of Fujian Medical University, Fujian Cancer Hospital, Fuzhou, 350014 Fujian China; 3 School of Nuclear Science and Technology, University of South China, Hengyang, 421001 Hunan China

**Keywords:** Tomotherapy, Hypothyroidism prediction, Radiomics, Dosiomics, Machine learning, Predictive markers, Cancer imaging, Head and neck cancer

## Abstract

The purpose of this study was to establish an integrated predictive model that combines clinical features, DVH, radiomics, and dosiomics features to predict RIHT in patients receiving tomotherapy for nasopharyngeal carcinoma. Data from 219 patients with nasopharyngeal carcinoma were randomly divided into a training cohort (n = 175) and a test cohort (n = 44) in an 8:2 ratio. RIHT is defined as serum thyroid-stimulating hormone (TSH) greater than 5.6 μU/mL, with or without a decrease in free thyroxine (FT4). Clinical features, 27 DVH features, 107 radiomics features and 107 dosiomics features were extracted for each case and included in the model construction. The least absolute shrinkage and selection operator (LASSO) regression method was used to select the most relevant features. The eXtreme Gradient Boosting (XGBoost) was then employed to train separate models using the selected features from clinical, DVH, radiomics and dosiomics data. Finally, a combined model incorporating all features was developed. The models were evaluated using receiver operating characteristic (ROC) curves and decision curve analysis. In the test cohort, the area under the receiver operating characteristic curve (AUC) for the clinical, DVH, radiomics, dosiomics and combined models were 0.798 (95% confidence interval [CI], 0.656–0.941), 0.673 (0.512–0.834), 0.714 (0.555–0.873), 0.698 (0.530–0.848) and 0.842 (0.724–0.960), respectively. The combined model exhibited higher AUC values compared to other models. The decision curve analysis demonstrated that the combined model had superior clinical utility within the threshold probability range of 1% to 79% when compared to the other models. This study has successfully developed a predictive model that combines multiple features. The performance of the combined model is superior to that of single-feature models, allowing for early prediction of RIHT in patients with nasopharyngeal carcinoma after tomotherapy.

## Introduction

Nasopharyngeal carcinoma is a malignant tumor that develops in the epithelium of the nasopharyngeal mucosa. It is relatively common in southern China, and its prevalence is on the rise^1,2^. Radiotherapy is widely regarded as a more effective treatment option for nasopharyngeal carcinoma. Specifically, intensity-modulated radiation therapy (IMRT) has emerged as the standard technique for radiation treatment in this case^3,4^. The increased adoption of IMRT, coupled with the systematic implementation of concurrent chemoradiotherapy, has greatly enhanced the treatment outcomes for nasopharyngeal carcinoma. As a result, the 5-year overall survival rate now stands at approximately 80%^5,6^.

The main adverse reactions in patients with nasopharyngeal carcinoma after radiotherapy and chemotherapy include dry mouth, hearing loss, skin atrophy, and thyroid dysfunction. The radiotherapy target area includes the nasopharynx and the lymphatic drainage area of the neck, where the thyroid gland is partially encompassed. Therefore, radiotherapy can easily affect thyroid hormone levels, leading to early concealed thyroid dysfunction that can impact patients’ quality of life and prognosis^7^. Studies have reported an incidence rate of 22–29% for radiotherapy-related thyroid dysfunction^8,9^. Thyroid dysfunction can manifest as fatigue, cold intolerance, dry skin, weight gain, constipation, or it can be asymptomatic, exerting varying degrees of impact on patients’ social function and quality of life^10^. Minimizing the occurrence of radiation-induced thyroid dysfunction has become a crucial consideration in the treatment strategy for nasopharyngeal carcinoma patients and remains one of the hot topics that need to be addressed.

Numerous studies have investigated factors associated with radiation-induced hypothyroidism (RIHT) in patients with nasopharyngeal carcinoma and other head and neck tumors. Tumor stage, gender, age, and baseline hematological parameters have been identified as demographic factors for radiation-induced hypothyroidism^11–15^. Many studies have found a clear correlation between thyroid dose and volume, which has a significant impact on the incidence of RIHT. Among them, Vx (referring to the percentage of thyroid volume exposed to a dose higher than xGy) is a dosimetric predictive parameter commonly used in many studies^16–21^. Additionally, researchers have developed NTCP models to predict RIHT and optimize the radiation dose for the thyroid based on risk factors^22–24^. However, relying solely on physical dose parameters from dose-volume histograms (DVH) may only provide partial information about dose distribution and lack information about voxel spatial relationships. It is important to understand that equivalent physical dose values can arise from distinct dose distributions with varying spatial relationships, potentially leading to different biological effects. Furthermore, the sensitivity of the thyroid gland to radiotherapy differs among patients, and the factors contributing to the development of RIHT are varied, thus necessitating individualized analysis.

In recent years, there has been a growing application of technologies such as artificial intelligence (AI) and machine learning in the field of identifying radiation-induced complications. Radiomics is a novel approach used to extract a multitude of quantitative features from medical images, including CT scans. These images are then thoroughly analyzed for diagnostic and prognostic purposes. By utilizing computer algorithms and machine learning techniques, medical images are converted into quantifiable data, allowing for the exploration of the extensive feature information embedded within them^25,26^. Similar to radiomics, dosiomics is a technique that automatically extracts quantitative features from the dose distribution matrix. Dosiomics features, compared to traditional DVH dose parameters, provide detailed spatial information about the three-dimensional dose distribution. These features effectively describe the impact of the dosage on the human body. Both radiomics and dosiomics have been employed to predict hematologic, lung, and esophageal toxicity. Previous research has strongly demonstrated that this approach greatly improves the predictive capability of the model. However, there is a scarcity of research reports on RIHT in patients with nasopharyngeal carcinoma. Ren has reported a predictive model based on dosiomics features, which exhibits superior performance compared to conventional dose-volume parameters^27^. However, their research has focused solely on single-type feature variables. Ritlumlert et al. developed a combined model that incorporates clinical features, dvh and radiomics features extracted from pre-radiotherapy CT scans, which significantly outperforms traditional models that only use single-type feature to predict RIHT^28^. The majority of research focuses on nasopharyngeal carcinoma patients undergoing IMRT or volumetric modulated arc therapy (VMAT) treatment; Nonetheless, there is limited research on RIHT in nasopharyngeal carcinoma patients undergoing tomotherapy.

The objective of this study was to establish an integrated predictive model that combines clinical features, DVH, radiomics, and dosiomics features to predict RIHT in patients receiving tomotherapy for nasopharyngeal carcinoma. This model serves to clinical oncologist practitioners in recognizing populations at high risk for RIHT development and facilitating the application of tailored interventions.

## Methods

### Patients

This study has been approved by the ethics committee of Fujian Cancer Hospital (ethics number: YKT2020-011-01) and all patients provided written informed consent prior to enrollment in the study. All methods were performed in accordance with the Declaration of Helsinki as well as relevant guidelines and regulations. The study included 219 patients diagnosed with nasopharyngeal carcinoma who underwent tomotherapy at Fujian Cancer Hospital from January 2017 to December 2020. The following clinical information was collected: patient age, gender, TNM stage, and the pre-treatment thyrotropin-stimulating hormone (TSH) levels. The patients were randomly divided into a training set (n = 175) and a test set (n = 44) in an 8:2 ratio. There were no statistically significant differences in clinical characteristics between the two groups of patients.

Inclusion criteria: (1) Patients with newly diagnosed nasopharyngeal carcinoma confirmed by pathology; (2) Patients in need of radical radiotherapy; (3) Patients aged between 18 and 70 years; (4) Patients with normal thyroid function and no underlying thyroid-related diseases; (5) Patients with a PS (ECOG criteria) score of 0 to 1.

Exclusion criteria: (1) Patients who underwent previous head and neck radiotherapy or thyroid surgery; (2) Patients with previous malignant tumors; (3) Patients with severe cardiovascular diseases or other underlying conditions that may affect the standard treatment of nasopharyngeal carcinoma. (4) Patients who did not have a complete follow-up result for thyroid function assessment.

### Thyroid function assessment

Before treatment, levels of total triiodothyronine (TT3), total thyroxine (TT4), free triiodothyronine (FT3), FT4, TSH, thyroglobulin antibody (TGAb), thyroid peroxidase antibody (TPOAb), and thyroglobulin (TG) are measured using chemiluminescence analysis to exclude underlying thyroid-related diseases. Thyroid hormone levels, such as TT3, TT4, FT3, and FT4, play a critical role in the regulation of metabolism and energy levels. TSH is secreted by the pituitary gland to oversee the thyroid’s hormone secretion. TGAb and TPOAb are linked to autoimmune thyroid disorders such as Hashimoto’s and Graves’ disease. TG is a protein crucial for thyroid hormone synthesis. During the follow-up before, at the end of, and after radiotherapy, levels of FT4, FT3, and TSH are determined using chemiluminescence analysis.

Follow-up visits consist of a combination of outpatient examinations and telephone communication. Within the first two years after completing treatment, follow-up visits occur every three months. From 3 to 5 years after treatment, monthly follow-up visits are conducted. During each follow-up visit, levels of FT4, FT3, and TSH are checked. The last follow-up in this study took place in June 2023, and the primary evaluation indicator was primary hypothyroidism (HT), defined as a serum TSH level greater than 5.6 μU/mL with or without a decrease in FT4 levels^29^. The occurrence time of RIHT was defined as the time interval between the end of radiotherapy and the first recorded abnormal TSH level.

### Image acquisition, contouring, and radiation dose calculation

Based on the 8th edition of the UICC/AJCC staging system^30^, patients in stage I undergo curative radiotherapy, stage II patients receive combined chemotherapy and radiotherapy, and stage III–IVB patients undergo combination therapy, all of which include radiation therapy. Positioning CT scans are acquired using the Philips Brilliance Big Bore CT. Patients are positioned supine and immobilized using thermoplastic masks and customized foam. The tube voltage is set at 120 kV, X-ray tube current is 225 mA, CT scan slice thickness is 3 mm, and the scan resolution is 512 × 512 pixels.

In accordance with the guidelines RTOG0225 and RTOG0615, experienced radiation oncologists with over 5 years of experience delineate GTV, CTV, and PTV target areas. The organs at risk, such as the thyroid, are separately delineated by two junior radiation oncologists, each with a minimum of 2 years of experience, and the final delineation is verified by senior radiation oncologists. The prescribed radiotherapy doses were as follows: GTV: 70–72.6 Gy/31–33 fractions, CTV1: 62–62.7 Gy/31–33 fractions, and for CTV2: 54.4–56.2 Gy/31–33 fractions. The dose limitation of organs at risk (OARs) listed in supplementary material Table S1.

All patients undergo intensity-modulated radiation therapy using the Accuray TomoHD helical tomotherapy system (Accuray Inc., Madison, Wisconsin) for treatment planning. The radiation energy used is 6 MV, the dose rate is 850 MU/min, and the dose calculation algorithm employed is the convolution/superposition (C/S) algorithm within the tumor treatment planning system. The voxel spatial resolution for dose calculation is 0.273 × 0.273 × 0.3 cm^3^. The primary objective of the treatment plan is to deliver sufficient and consistent dose to the planning target volume (PTV) while minimizing radiation exposure to OARs.

### Radiomics and dosiomics features extraction

Feature extraction is the process of calculating a large number of specific parameters from a region of interest (ROI). This study utilizes the Pyradiomics open-source package, which is based on the Python 3.7 platform, to extract radiomic and dosiomics features. The extracted radiomic features are categorized into three groups: first-order statistical features, shape features, and texture features. First-order statistical features indicate changes in symmetry, uniformity, and local intensity distribution within the measured ROI region. Shape features provide quantitative descriptions of the three-dimensional size and morphological information of the ROI region. Texture features reflect the spatial arrangement of grayscale values within the ROI region. For detailed descriptions of each feature type, please refer to the official Pyradiomics documentation^31^. A total of 107 radiomic features and 107 dosiomics features were extracted from each patient, respectively.

### Features selection and model building of radiomics and dosiomics

The Kolmogorov–Smirnov test is utilized to assess the normality of feature distribution. When the computed p-value of the test statistic is below the predefined significance threshold (typically 0.05), the null hypothesis is rejected, signifying a departure from normal distribution. In contrast, if the p-value exceeds the significance level, the null hypothesis is upheld, indicating conformity to a normal distribution. Then, we applied the Student’s *t*-test to analyze features that follow a normal distribution, and the Mann–Whitney *U* test to analyze features that do not follow a normal distribution. The null hypothesis for the Student’s *t*-test states no significant difference between the means of the two groups compared for a specific feature, while the null hypothesis for the Mann–Whitney *U* test asserts no significant difference between the distributions of the two groups compared for a particular feature. When the p-value from the Student’s *t*-test or Mann–Whitney *U* test exceeds 0.05 for two sets of features, there is no significant distinction between them, hence one feature ought to be randomly removed. Additionally, we used the Spearman rank correlation coefficient to measure the correlation between highly correlation features. If the correlation coefficient between any two features exceeded 0.9, we retained only one of the features. To preserve the descriptive ability of the features, we implemented a greedy recursive feature elimination strategy, eliminating the feature with the highest correlation in each iteration. Lastly, we employed the Least Absolute Shrinkage and Selection Operator (LASSO) regression model to construct features in the dataset. By adjusting the regularization weight lambda, the LASSO regression model sets all regression coefficients to zero, resulting in many coefficients of irrelevant features becoming zero. To determine the optimal lambda value that minimizes the standardized error and achieves the lowest cross-validation error, we performed tenfold cross-validation. The remaining non-zero coefficient features were combined with radiomics features for the regression model. By linearly combining the remaining features and their model coefficient weights, we obtained a radiomics score for each patient. We implemented the LASSO regression model using the Python scikit-learn library. After Lasso feature screening, we input the final features into the XGBoost machine learning models and so forth for risk model construction. The fundamental concept of the XGBoost model is to integrate numerous weak classifiers (decision trees) to form a robust classifier. Each decision tree is trained on the residuals of the preceding tree, progressively diminishing the residuals by iteratively optimizing the loss function. Simultaneously, the model mitigates the risk of overfitting by managing the trees’ complexity and implementing regularization terms. In this study, the XGBoost is fed with diverse feature types as input, and it produces the probability value of RIHT occurrence as output. Here, we adopt fivefold cross-verification to obtain the final rad signature.

### Clinical and DVH model building

The study incorporates age, gender, T stage (1, 2, 3, 4), N stage (0, 1, 2), TNM stage (1, 2, 3, 4), and the pre-treatment TSH value as clinically relevant features. The DVH features consist of the max dose (D_max_), the min dose (D_min_), the mean dose (D_mean_), i cubic centimeter of the maximum dose (D_icc_, with i ranging from 1 to 10 at an interval of 1), and percentage of volume that has received at least j Gy radiation (V_jGy_ with j ranging from 5 to 65 at an interval of 5), resulting in a total of 23 thyroid volume features. The construction process of clinical signatures closely resembles that of rad signatures. Initially, baseline statistics (the p-value from the Student’s *t*-test or Mann–Whitney *U* test) were used to select the features for constructing the clinical characteristics. Furthermore, the same machine learning model was utilized in the construction process of clinical and DVH signatures. To ensure fairness in comparison, fivefold cross-validation and a fixed experimental queue were adopted.

### Combined model

**Figure 1:**
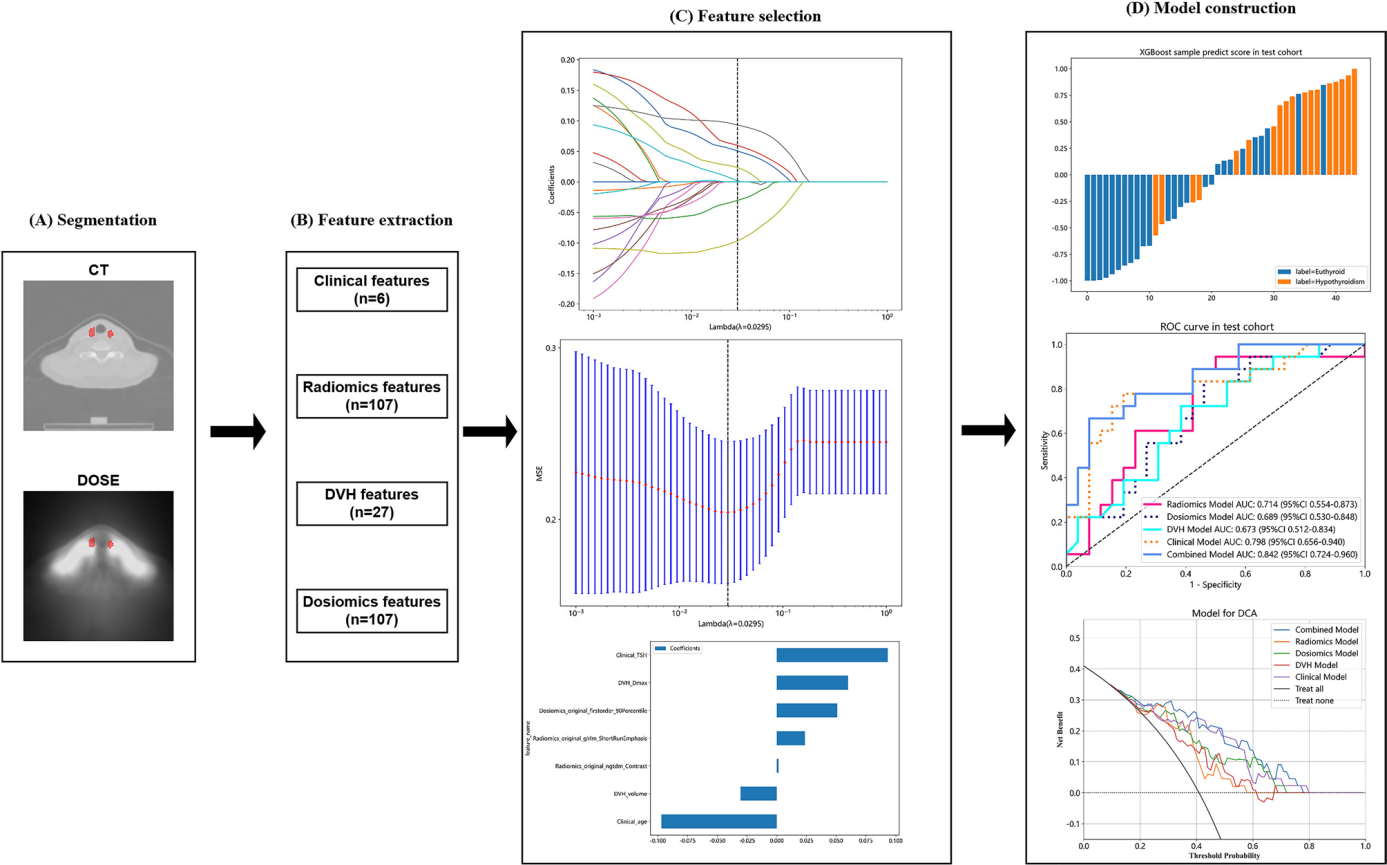
The workflow of the combined model construction.

According to Fig. 1, the clinical features, DVH parameters, radiomics features, and dosiomics features were integrated as inputs to the composite model. Additionally, valuable features were selected using Student’s *t*-test or Mann–Whitney *U* test and LASSO regression, and then inputted into the XGBoost machine learning model for thyroid toxicity prediction. Similarly, fivefold cross-validation and a fixed experimental queue were adopted.

### Statistical analysis

In order to assess the diagnostic performance, we conducted tests in an experimental cohort. In order to examine the equivalence of patient attributes between different cohorts, we employed independent *t*-tests to analyze normally distributed data and utilized the Mann–Whitney *U* test to represent non-normally distributed data using medians (interquartile ranges). For categorical variables, we used the Chi-square test for analysis. Additionally, we evaluated the predictive performance of the three models using receiver operating characteristic (ROC) curves, where we calculated the area under the ROC curve (AUC), as well as the trade-off between sensitivity and specificity at the maximum Youden index. Furthermore, we evaluated the performance of these three models in the training and testing cohorts and assessed the clinical utility of the radiomics-clinical model using decision curve analysis (DCA).

Statistical analyses were performed using SPSS (version 21.0; IBM Corp.) and the “One-key AI” platform (https://www.medai.icu), which is based on Pytorch 1.8.0. Statistical significance was defined as a two-sided p-value ≤ 0.05.

## Results

### Patient characteristics

The baseline characteristics of patients in the training and test cohorts are presented in Table 1. The median follow-up duration of all patients was 28 months (range 3–63 months). The average age of the patients in the training cohort was 46.59 ± 13.08 years, including 125 males (71.4%) and 50 females (28.6%). The average age of the patients in the test cohort was 46.68 ± 13.61 years, including 35 males (79.5%) and 9 females (20.5%). Among them, those in the training cohort and the test cohort had no significant differences in clinical baseline characteristics (sex, age, T stage, N stage, clinical stage, etc.).

**Table 1:** Baseline characteristics of patients in the training and test cohorts.

Characteristic	Training cohort (n = 175)	Test cohort (n = 44)	P
Age, years	46.59 ± 13.08	46.68 ± 13.61	0.859
Pre-treatment TSH, µIU/L	1.70 ± 0.96	1.69 ± 0.94	0.975
Gender			0.278
Male	125 (71.4%)	35 (79.5%)	
Female	50 (28.6%)	9 (20.5%)	
T stage (UICC/AJCC 7th edition)			0.787
T_1_	39 (22.3%)	12 (27.3%)	
T_2_	29 (16.6%)	6 (13.6%)	
T_3_	62 (35.4%)	17 (38.6%)	
T_4_	45 (25.7%)	9 (20.5%)	
N stage (UICC/AJCC 7th edition)			0.147
N_0_	21 (12.0%)	9 (20.5%)	
N_1_	75 (42.8%)	22 (50.0%)	
N_2_	47 (26.9%)	10 (22.7%)	
N_3_	32 (18.3%)	3 (6.8%)	
Clinical TNM stages (UICC/AJCC 7th edition)			0.235
I	8 (4.6%)	5 (11.4%)	
II	36 (20.5%)	9 (20.5%)	
III	63 (36.0%)	18 (40.8%)	
IV	68 (38.9%)	12 (27.3%)	
Outcome			0.912
Hypothyroidism	70 (40.0%)	18 (40.9%)	
Euthyroid	105 (60.0%)	26 (59.1%)	

### Clinical model

The features utilized to develop the clinical model were determined based on the p-value (≤ 0.05) of the characteristics within the training cohort. Only baseline age and pre-treatment TSH met this criterion. These two characteristics were also used in constructing the Clinical Signature.

The clinical model demonstrated an AUC of 0.963 (95% CI 0.939–0.987) with balanced sensitivity and specificity of 0.884 and 0.913, respectively, in the training cohort. In the test cohort, the AUC was 0.798 (95% CI 0.656–0.940), and the sensitivity and specificity were 0.722 and 0.808, respectively (Table 3).

### DVH model

Consistent with the clinical model, the DVH model employed a similar approach to filter features. Dmax, D1cc, V60, and volume were selected as meaningful features for constructing the DVH model.

The DVH model demonstrated an AUC of 0.988 (95% CI 0.978–0.999) with balanced sensitivity and specificity of 0.942 and 0.951, respectively, in the training cohort. In the test cohort, the AUC was 0.673 (95% CI 0.512–0.834), and the sensitivity and specificity were 0.722 and 0.615, respectively (Table 3).

### Radiomics model

After the selection process, a total of 4 features with non-zero coefficient values were retained. These four features are: shape voxel volume, shape maximum 3D diameter, shape minor axis length and gray level run length matrix run variance. The radiomics signature was constructed based on the coefficient values of the selected features.

The selected features were utilized to construct the radiomics model. In the training cohort, the model demonstrated an AUC of 0.997 (95% CI 0.993–1.000), with balanced sensitivity and specificity of 0.943 and 0.990, respectively. In the test cohort, the AUC was 0.714 (95% CI 0.545–0.873), and the sensitivity and specificity were 0.556 and 0.769, respectively (Table 3).

### Dosiomics model

Consistent with the radiomics model, the dosiomics model employed a similar approach to filter features. Similarly, four features were selected to establish the dosiomics model. These four features are: firstorder maximum, firstorder 90th percentile, gray level co-occurrence matrix maximal correlation coefficient and shape voxel volume.

The selected features were utilized to construct the radiomics model. In the training cohort, the model demonstrated an AUC of 0.993 (95% CI 0.986–1.000), with balanced sensitivity and specificity of 0.971 and 0.952, respectively. In the test cohort, the AUC was 0.698 (95% CI 0.530–0.848), and the sensitivity and specificity were 0.556 and 0.615, respectively (Table 3).

### Combined model

**Figure 2:**
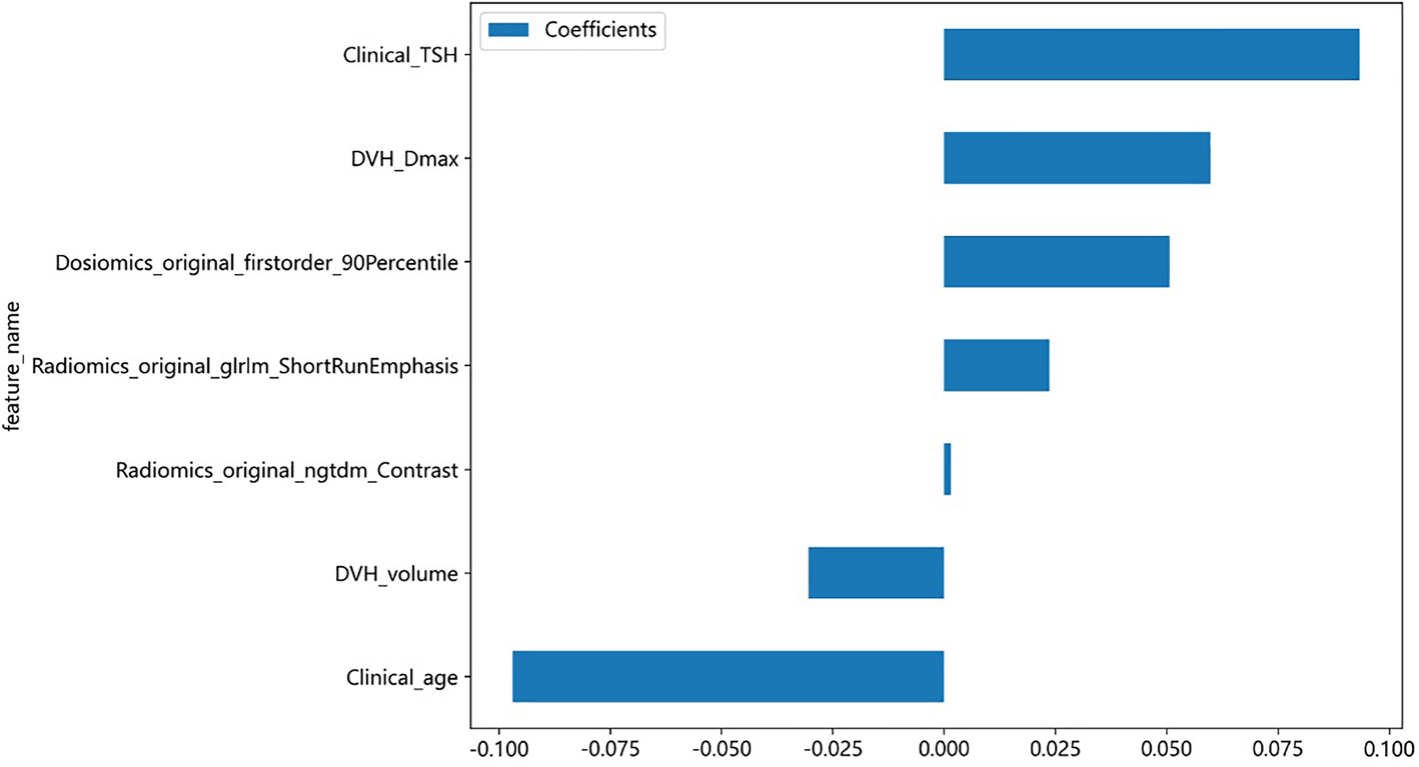
The histogram of the coefficients of the selected features. *GLRLM* gray-level run length matrix, *NGTDM* neighborhood gray-tone difference matrix.

Age, pre-treatment TSH , thyroid volume, Dmax, radiomics original gray level run length matrix short run emphasis, radiomics original neighbouring gray tone difference matrix contrast, and dosiomics original firstorder 90th percentile were selected as the most valuable features in the combination model, and their feature coefficient histograms and feature importance are shown in the Fig. 2 and Table 2.

**Table 2:** Feature importance of the combined model.

Features	Weights
Clinical_age	0.22654682
DVH_Dmax	0.16664952
Clinical_TSH	0.16540626
DVH_volume	0.12443488
Radiomics_original_ngtdm_Contrast	0.11686287
Dosiomics_original_firstorder_90Percentile	0.113821395
Radiomics_original_glrlm_ShortRunEmphasis	0.0862782

**Figure 3:**
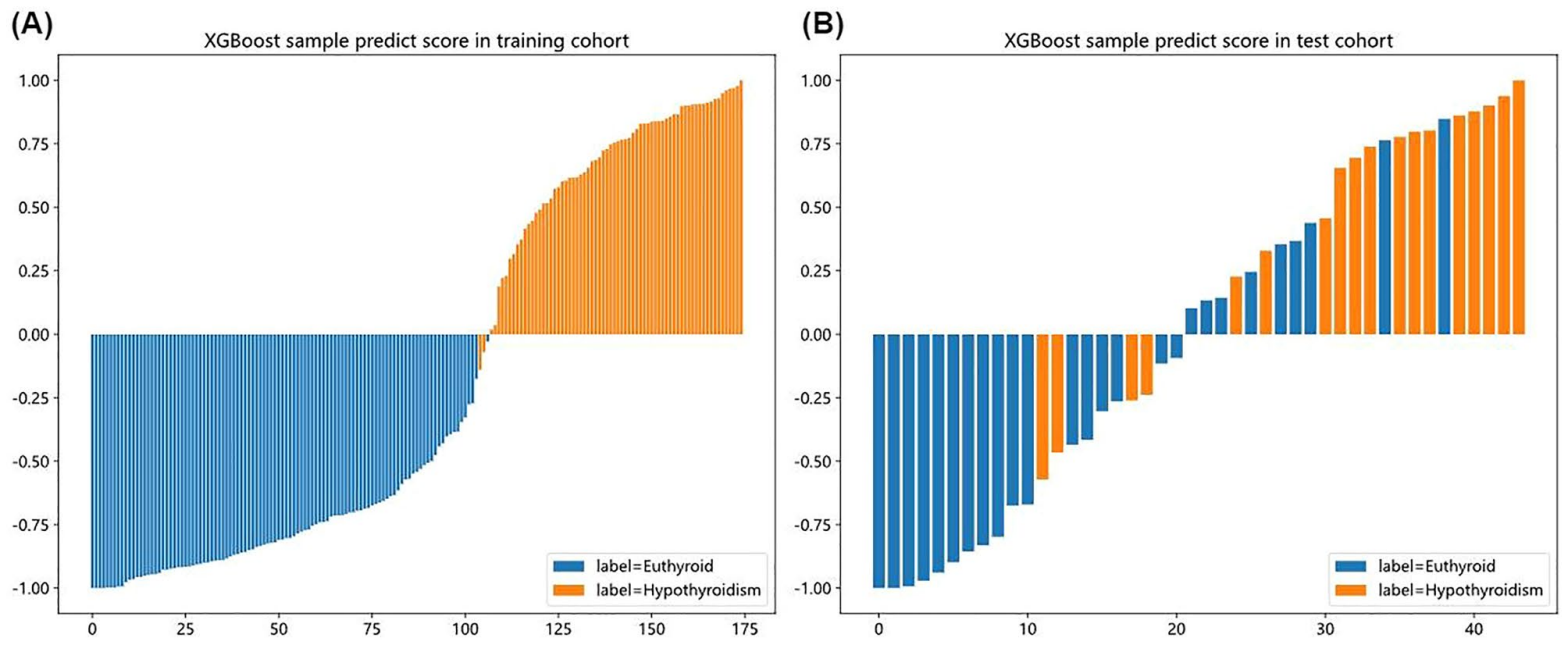
Waterfall chart reflects the performance of the training cohort (**A**) and test cohort (**B**) in the prediction of RIHT.

In the training cohort, the establishment model exhibited an area under the curve (AUC) value of 1.000 (95% CI 0.999–1.000), with equally balanced sensitivity (1.000) and specificity (0.990). The test cohort yielded an AUC of 0.842 (95% CI 0.724–0.960), with a sensitivity of 0.778 and a specificity of 0.615 (Table 3). Waterfall chart reflects the predict score of the training cohort and test cohort in the prediction of RIHT (Fig. 3).

**Table 3:** Predictive performance of five models in the training cohort and test cohort.

Model	Dataset	Accuracy	AUC	95% CI	Sensitivity	Specificity	PPV	NPV	Precision	Recall	F1	Threshold
DVH	Train	0.948	0.988	0.978–0.999	0.942	0.951	0.929	0.961	0.929	0.942	0.935	0.463
	Test	0.659	0.673	0.512–0.834	0.722	0.615	0.565	0.762	0.565	0.722	0.634	
Dosiomics	Train	0.960	0.993	0.986–1.000	0.971	0.952	0.932	0.980	0.932	0.971	0.951	0.420
	Test	0.591	0.689	0.530–0.848	0.556	0.615	0.500	0.667	0.500	0.556	0.526	
Radiomics	Train	0.971	0.997	0.993–1.000	0.943	0.990	0.985	0.963	0.985	0.943	0.964	0.513
	Test	0.682	0.714	0.555–0.873	0.556	0.769	0.625	0.714	0.625	0.556	0.503	
Clinical	Train	0.901	0.963	0.939–0.987	0.884	0.913	0.871	0.922	0.871	0.884	0.878	0.418
	Test	0.773	0.798	0.656–0.941	0.722	0.808	0.722	0.828	0.722	0.722	0.808	
Combined	Train	0.994	1.000	0.999–1.000	1.000	0.990	0.986	1.000	0.986	1.000	0.993	0.428
	Test	0.682	0.842	0.724–0.960	0.778	0.615	0.583	0.800	0.583	0.778	0.667

**Figure 4:**
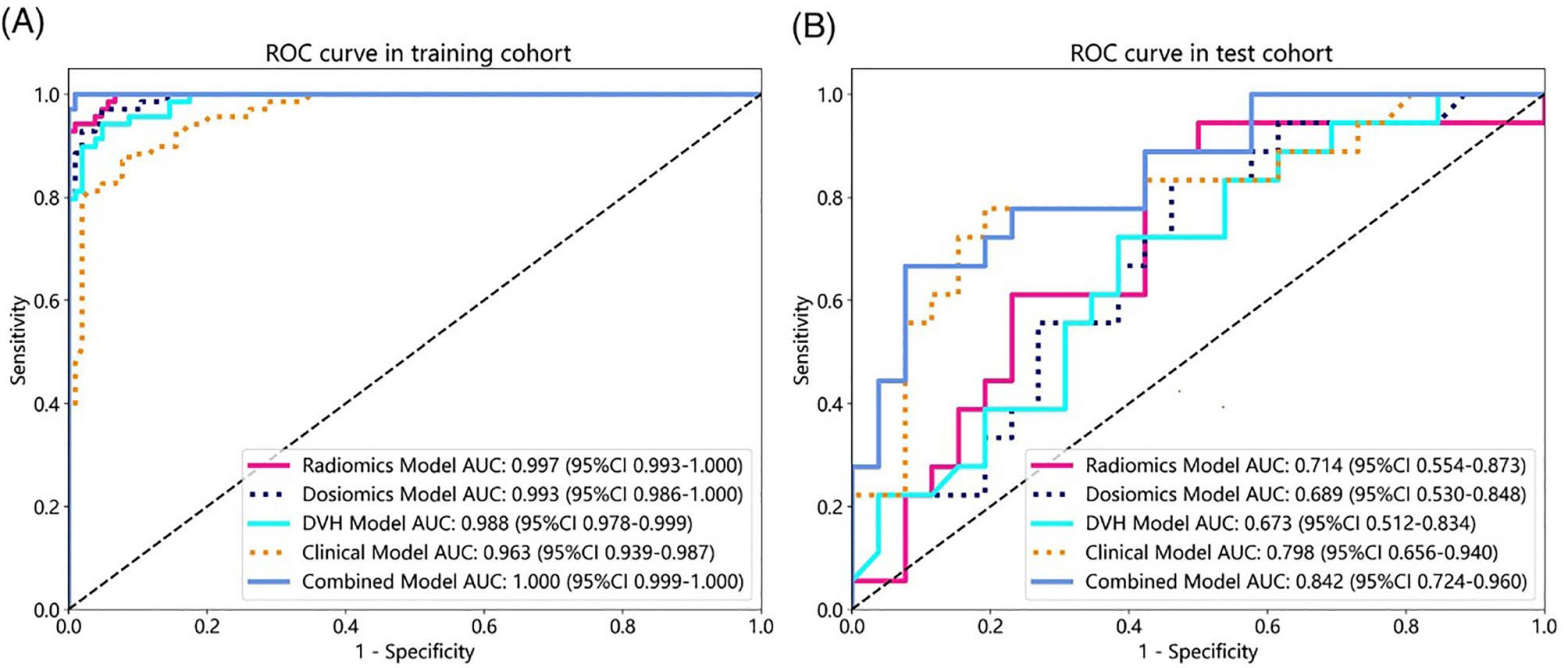
ROC curves of the radiomics model, dosiomics model, DVH model, clinical model and combined model in the training cohort (**A**) and test cohort (**B**). *ROC* receiver operating characteristic.

**Figure 5:**
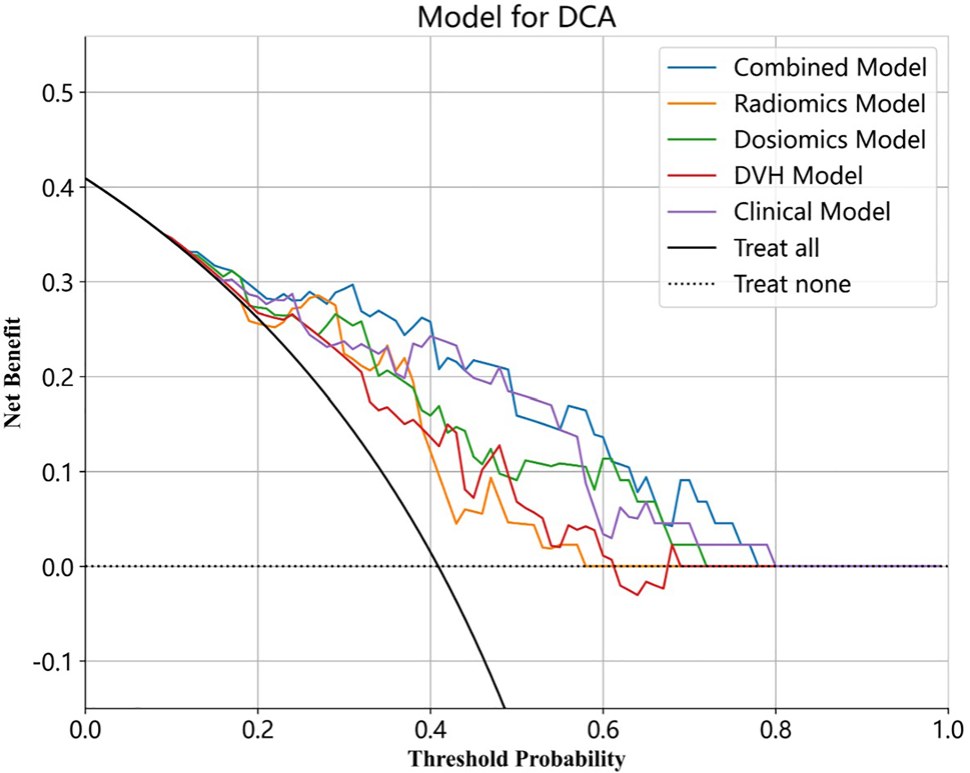
DCA of the radiomics model, dosiomics model, DVH model, clinical model and combined model in the test cohort.

The calibration curve illustrated that the RIHT predicted by the combined model closely matched the actual results in both datasets. Additionally, the decision curve analysis (DCA) demonstrated improvement in the combined model for both datasets (Fig. 4). This finding indicated that within a threshold probability range of 1% to 79%, the combined model outperformed the other models in terms of benefits (Fig. 5).

## Discussion

In this study, we developed a novel combination model that incorporates clinical parameters, DVH parameters, radiomics features, and dosiomics features to predict RIHT in patients with nasopharyngeal carcinoma undergoing radiotherapy. The results demonstrated that the combination model achieved AUC values of 1.000 and 0.842 in the training and testing groups, respectively. Compared to single feature models, the combination model displayed better discriminative ability and goodness of fit, indicating better diagnostic performance. Age, pre-treatment TSH, thyroid volume, Dmax, radiomics original gray level run length matrix short run emphasis, radiomics original neighbouring gray tone difference matrix contrast, and dosiomics original firstorder 90th percentile can serve as reliable predictive features for RIHT in patients with nasopharyngeal carcinoma undergoing tomotherapy.

RIHT is a frequent complication in radiotherapy for nasopharyngeal carcinoma due to the close proximity or overlap of the radiation target area with the nearby thyroid gland, often accompanied by cervical lymph node metastasis at initial diagnosis. Consequently, the thyroid gland unavoidably receives radiation during treatment. According to Zhai et al.’s report, the incidence of RIHT in nasopharyngeal carcinoma patients within 3 and 5 years after radiotherapy was 39.4% and 49.1%, respectively. For this study, we included a cohort of 219 patients, and the overall incidence of RIHT during the follow-up period was 40.2%, consistent with the findings of Zhai et al.^32^.

In previous studies on the prediction of RIHT for nasopharyngeal carcinoma, age has been considered a major clinical risk factor. Zhai et al. found that age independently affected RIHT after IMRT, with younger patients being more prone to developing hypothyroidism^32^. Similarly, Wu et al. also found in their study that age was one of the factors influencing the occurrence of hypothyroidism, suggesting that the thyroid gland’s sensitivity to radiation decreases with age^13^. Furthermore, studies have shown that female patients have a higher risk of developing RIHT. Hancock et al. observed an increased risk of hypothyroidism occurrence in females^33^. However, Diaz et al. and Wu et al. stated that gender does not play a role in the development of hypothyroidism. They suggested that this may be due to the smaller thyroid volume in females compared to males, and the gender effect could be confounded by differences in thyroid volume^13,14^. In this study, age and pre-treatment TSH were identified as important predictive factors for RIHT. However, it is not a routine practice to perform serum thyroid hormone testing in nasopharyngeal carcinoma patients before and after radiotherapy during follow-up. Therefore, it is recommended to conduct baseline thyroid function testing before starting radiotherapy and regularly monitor it to detect potential temporary functional impairments and the risk of eventually developing permanent hypothyroidism early on.

In the treatment of patients with nasopharyngeal carcinoma, the neck is typically exposed to radiation doses ranging from 50–70 Gy. However, the exact threshold dose of radiation that causes direct damage to the thyroid remains uncertain. Recent studies suggest that the dose of radiation received by the thyroid gland is a crucial factor in the development of RIHT. Nonetheless, the findings of different studies are inconsistent. Akgun et al. reported a significant correlation between thyroid volume, V30 (the proportion of the volume receiving a dose of 30 Gy or higher), average thyroid dose, and the occurrence of RIHT^19^. Stella et al. recommend specific targets for radiation therapy planning, including D50% < 50 Gy, V50 < 50%, and average dose < 54.58 Gy, in order to reduce the risk of RIHT^11^. However, some researchers argue that the toxic effects of different radiation doses on the thyroid are still unclear. They propose using dose-volume histogram (DVH) curves as a reference for thyroid constraints, rather than relying solely on a specific point on the DVH curve. Huang et al. conducted a retrospective analysis of 345 patients with nasopharyngeal carcinoma treated with intensity-modulated radiation therapy (IMRT) and found that patients in Group (V25 ≤ 60%, V35 ≤ 55%, V45 ≤ 45%) had a significantly lower incidence of RIHT (13.2% vs. 25.8%) and were independently associated with decreased risk of developing radiation-induced hypothyroidism^12^. Ren et al. argue that traditional dose-volume factors represent discrete points on the DVH curve and provide insufficient information regarding dose distribution, including intensity, shape, size, and distribution of different doses^27^. Their study demonstrated that dose-volume models were superior to DVH models in predicting RIHT in nasopharyngeal carcinoma (AUC value: 0.70 vs. 0.61). Ritlumlert et al. developed a combined model that incorporates clinical features, dvh and radiomics features extracted from pre-radiotherapy CT scans, which significantly outperforms traditional models that only use single-type feature to predict RIHT (AUC value: 0.81)^28^.

Compared to previous studies, this study added more types of features to predict RIHT in nasopharyngeal carcinoma patients undergoing tomotherapy. The results revealed that the combined model outperformed single—feature models, exhibiting the highest AUC value, and the lower and upper limits of the 95% CI were higher than other models. The combined model exhibits the higher sensitivity, effectively mitigating the likelihood of overlooking detections in RIHT. In addition, the clinical model also performed well, achieving an AUC value of 0.798. Analysis of Table 2 highlights that age and TSH clinical features carry substantial weight in the combination model, constituting about 39% of all features. This suggests that clinical model attributes possess greater predictive significance for RIHT compared to DVH, radiomics and dosiomics features, yielding favorable predictive outcomes. Furthermore, our study indicate that the dosiomics model surpasses DVH models, consistent with the observations by Ren et al.^27^. Dosiomics offers comprehensive insights into dose distribution size, shape, and pattern, enhancing predictive accuracy beyond discrete DVH features. Additionally, our study demonstrates the superior performance of radiomics models over dosiomics models in prediction RIHT, underscoring the valuable predictive potential of deep features extracted from CT images. To conclude, the combined model estimates the likelihood of RIHT by amalgamating characteristics from CT images, dose-volume attributes from 3D dose maps, point dose parameters from DVH curves, along with patient age and pre-treatment TSH levels. This integrated approach aids in predicting RIHT occurrence prior to tomotherapy in nasopharyngeal cancer patients, thereby assisting clinicians in identifying high-risk individuals for targeted interventions to enhance patient quality of life.

Our study has some limitations. Firstly, the model is only applicable to nasopharyngeal carcinoma patients receiving tomotherapy, and further research is needed for other radiation techniques such as IMRT, VMAT, and three dimensional conformal radiotherapy (3D-CRT). Secondly, this study only included 219 cases, which has a relatively small sample size and lacks validation from external centers. Thirdly, we did not adopt more advanced techniques such as deep learning methods. In future research, we will increase the sample size and diversity, introduce data from external centers for further validation, and incorporate deep learning methods to improve the predictive performance of the model.

## Conclusion

This study established a combined predictive model based on clinical features, DVH parameters, radiomics and dosiomics features to predict the likelihood of RIHT in nasopharyngeal carcinoma patients undergoing radiotherapy. The model also has the potential to assist in identifying potential RIHT patients and implementing preventative measures.

### Supplementary Information

Supplementary Information.

## Data Availability

The datasets used and/or analysed during the current study available from the corresponding author on reasonable request.
