# Supplementary material for: A machine learning approach for predicting radiation-induced hypothyroidism in patients with nasopharyngeal carcinoma undergoing tomotherapy
Source: Sci Rep. 2024 Apr 10;14:8436. doi: 10.1038/s41598-024-59249-3 (PMC11006930; doi:10.1038/s41598-024-59249-3)
Supplement: Supplementary file 1 — Supplementary Information. [file 41598_2024_59249_MOESM1_ESM.docx]

**Supplementary Information**

**Table S1. The criteria of OARs**

| OARs | Criteria |
| --- | --- |
| Left/right lens | Dmax < 8 Gy |
| Left/right optic nerves | Dmax < 54 Gy |
| Optic chiasm | Dmax < 54 Gy |
| Brainstem | Dmax < 54 Gy |
| Spinal cord | Dmax < 45 Gy |
| Left/right parotid | Dmean < 30 Gy  V30 < 50% |
| Pituitary | Dmax < 54 Gy |
| Thyroid | V40 < 80% |

**Table S2. Cross validation of the training cohort**

| Models | K-Fold | AUC |
| --- | --- | --- |
| Combine model | 1 | **0.864** |
|  | 2 | 0.578 |
|  | 3 | 0.810 |
|  | 4 | 0.663 |
|  | 5 | 0.738 |
| Radiomics model | 1 | **0.752** |
|  | 2 | 0.645 |
|  | 3 | 0.713 |
|  | 4 | 0.703 |
|  | 5 | 0.604 |
| Dosiomics model | 1 | 0.668 |
|  | 2 | 0.633 |
|  | 3 | 0.609 |
|  | 4 | **0.721** |
|  | 5 | 0.711 |
| DVH model | 1 | 0.517 |
|  | 2 | 0.724 |
|  | 3 | 0.684 |
|  | 4 | **0.768** |
|  | 5 | 0.645 |
| Clinical model | 1 | 0.651 |
|  | 2 | 0.570 |
|  | 3 | **0.779** |
|  | 4 | 0.595 |
|  | 5 | 0.559 |


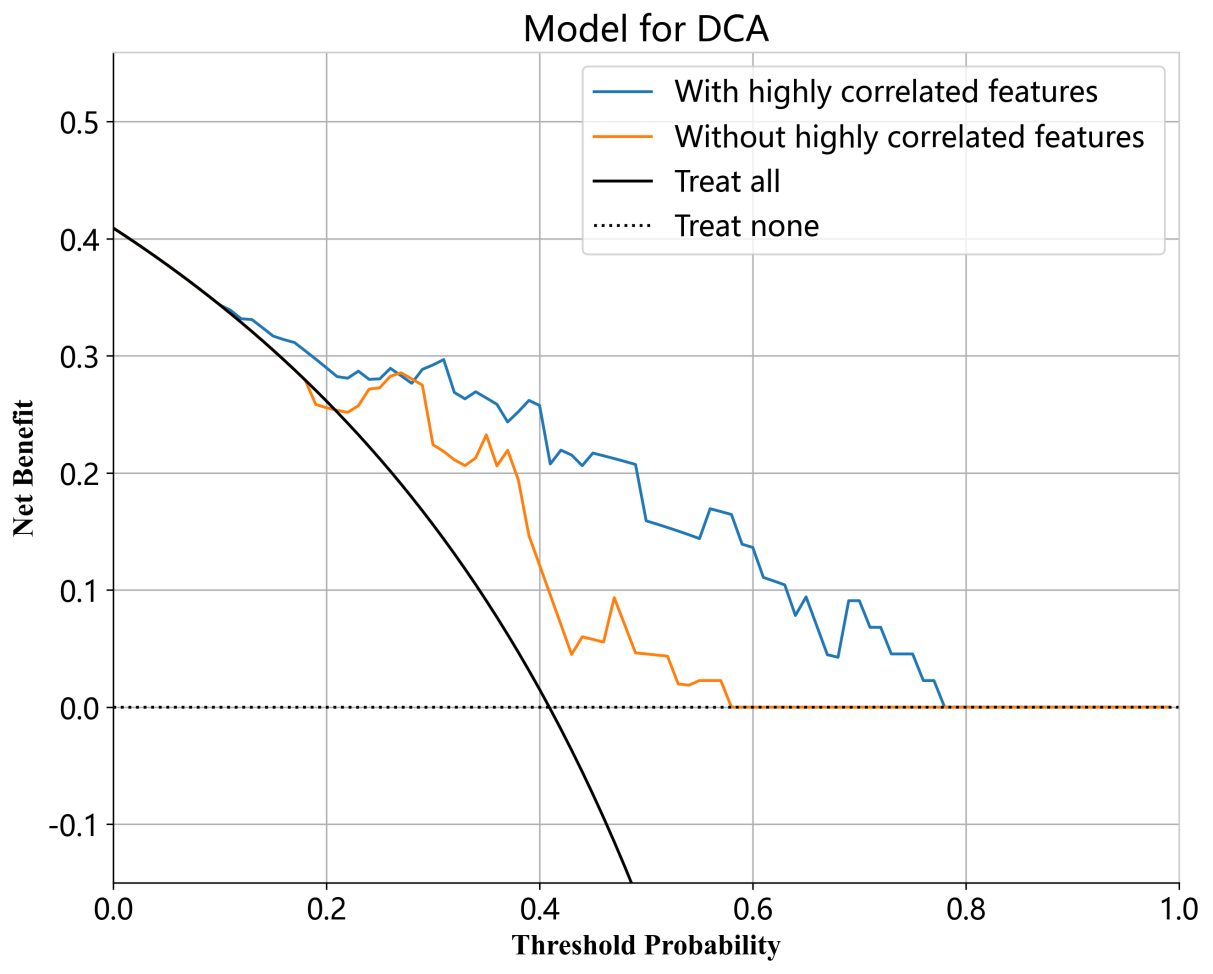


**Fig S1.** DCA of models with or without highly correlated features in the test cohort.
